# Intracardiac Echocardiography–Guided Implantation of Aveir VR Leadless Pacemaker

**DOI:** 10.1002/joa3.70303

**Published:** 2026-02-16

**Authors:** Kazuhisa Matsumoto, Naomichi Tanaka, Hitoshi Mori, Yoshifumi Ikeda, Ritsushi Kato

**Affiliations:** ^1^ Department of Cardiology Saitama Medical University International Medical Center Hidaka Japan

**Keywords:** Aveir VR, CIED infection, contrast allergy, intracardiac echocardiography, leadless pacemaker

## Abstract

Intracardiac echocardiography (ICE) enabled safe, contrast‐free implantation of an Aveir VR leadless pacemaker in a high‐risk patient with severe contrast allergy and prior device infection, allowing real‐time assessment of right ventricular anatomy and myocardial wall thickness to minimize perforation risk.

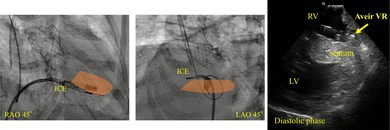

Leadless pacemaker (LP) plays an important role in the management of brady‐arrhythmias, particularly in patients who are at high risk of CIED infection. LPs are characterized by a high procedural success rate and a low incidence of complications with up to 50% reduction compared with a conventional transvenous pacemaker (TVPM) [1, 2]. However, cardiac perforation remains one of the most serious complications. It often requires surgical intervention, leading to be life‐threatening [3]. For this reason, delineation of right ventricular (RV) anatomy using contrast agents is important during implantation. However, the management of LP implantation in patients with severe contrast allergy or renal dysfunction who are high risk for contrast exposure has recently become an important clinical issue. Here, we report a case in which intracardiac echocardiography (ICE) was utilized to visualize RV anatomy and enable the safe implantation of LP in a patient with a severe contrast allergy.

A 72‐year‐old woman with a history of systemic lupus erythematosus treated with long‐term corticosteroids (prednisone 17.5 mg/day) had previously undergone transcatheter aortic valve implantation (TAVI) for severe aortic stenosis. During pre‐procedural contrast‐enhanced CT, she developed cardiopulmonary arrest due to a severe contrast allergy. She subsequently developed complete atrioventricular block and received a TVPM, chosen to avoid contrast use. Two years later, she was hospitalized with methicillin‐resistant *Staphylococcus aureus* (MRSA) bacteremia, which failed to clear despite antibiotic therapy, necessitating referral to our hospital for pacemaker lead extraction. After admission, daptomycin therapy was initiated. Complete lead removal was achieved successfully, and semi‐permanent pacing was performed using a permanent lead connected to an external device via the right internal jugular vein. Blood cultures were confirmed to be negative 1 week prior to the planned procedure, and LP implantation was scheduled on day 26. Daptomycin therapy was continued throughout the peri‐procedural period. Considering the possibility of reinfection and the need for device extraction, Aveir VR (Abbott, IL, USA), which has a dedicated retrieval tool, was selected. Moreover, because of the severe contrast allergy, ICE guidance was employed without use of contrast.

The procedure was performed under deep sedation. A 27‐Fr Aveir VR introducer was inserted via the right femoral vein, and a 10‐Fr long sheath for ViewFlex Xtra (Abbott, IL, USA) via the left femoral vein. Arterial pressure was monitored via the left femoral artery. ICE was advanced into the RV and adjusted to provide a view encompassing both ventricles. Although the Aveir VR initially appeared to be oriented toward the septum (Figure 1), ICE revealed that the device was actually directed toward the apical free wall. It was not feasible to further direct the catheter toward the septum. However, ICE also revealed a wall thickness of 8 mm at the apical free wall, which was greater than the length of the coiled spring (1.63 mm) of Aveir VR (Figure 2, Video S1). Based on this, screw‐in at the free wall was considered feasible. Pre‐mapping demonstrated a pacing threshold of 1.0 V at 0.4 ms, impedance of 350 Ω, and adequate current of injury (COI) was clearly observed. Impedance progressively increased with rotation (1 turn: 480 Ω, 1.5 turn: 530 Ω). Finally, the threshold was 1.25 V at 0.4 ms and impedance was 540 Ω, with stable parameters. The procedure was completed without complications such as cardiac tamponade. Prior to discharge (postoperative day 5), the pacing threshold was 0.5 V at 0.4 ms, and the impedance was 410 Ω. The patient had an uneventful postoperative course without worsening of infection and was subsequently discharged.

**FIGURE 1:**
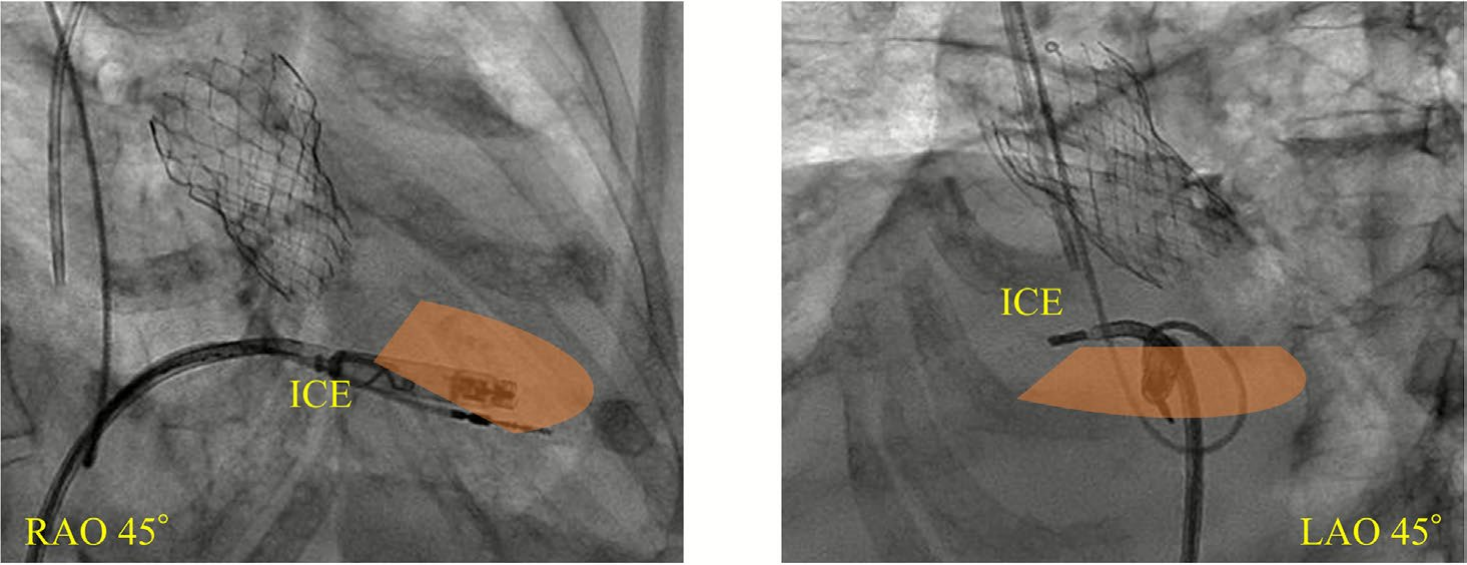
Fluoroscopic image showing catheter positioning during implantation. The orange‐colored area represents the region visualized by ICE.

**FIGURE 2:**
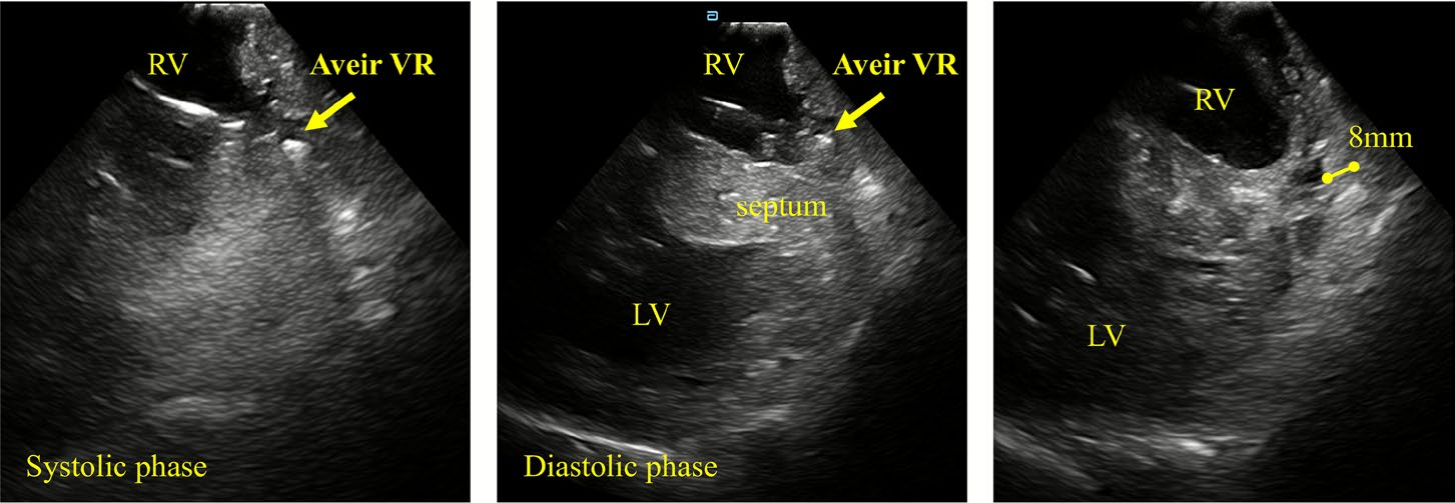
Intracardiac echocardiography image during implantation. Aveir VR was directed toward the apical free wall, where the myocardial wall thickness was 8 mm.

LP is an important therapeutic option for patients with bradyarrhythmia and prior device infection. There is growing anticipation for the development of safer implantation techniques for LPs, whose use is expected to continue increasing in the future. In this case, the patient had persistent MRSA bacteremia following TVPM and also had a history of life‐threatening contrast allergy, leaving ICE‐guided LP implantation as the most feasible approach. A unique feature of this case was the placement of the ViewFlex Xtra ICE catheter at the RV lateral side to visualize the right ventricle–interventricular septum–left ventricle around apex in a single view, which we considered to approximate a horizontal imaging plane. RV wall thickness was measured during diastole, and because minor variations can occur due to cardiac motion, measurements were repeated at least five times. Although RV wall thickness is generally reported to be approximately 2–5 mm, it appeared thicker in this patient. Given that left ventricular wall thickness was also mildly increased, the presence of underlying hypertensive remodeling was considered a possible contributing factor. By comparing the echogenicity of the measured region with that of the interventricular septum and left ventricular myocardium, we judged that the measurement reflected RV myocardial tissue up to the assessed depth (Figure 2, right panel). However, the right ventricular cavity has a complex, trabeculated structure, and myocardial thickness varies across different cross‐sections. Precise exclusion of epicardial fat or pericardial structures is challenging, which represents a limitation of echo‐guided implantation. Importantly, by observing the screwing process in real time using ICE, we were able to assess for potentially hazardous catheter behavior suggestive of myocardial perforation and avoid regions with critically thin myocardium. Although the apical free wall is generally not a recommended site for Aveir VR implantation, septal positioning was difficult in this case. This highlights the importance of wall thickness assessment when considering non‐septal fixation. Previous reports have described echocardiographic guidance for LP implantation. Yarmohammadi et al. reported successful Micra (Medtronic, Minneapolis, MN) implantation using three‐dimensional transthoracic echocardiography [4], noting advantages in cost and wide visualization from atrium to ventricle, although detailed apical assessment remained challenging. Chatani, et al. reported Micra implantation under right atrial ICE guidance without contrast [5], which was effective for assessing septal positioning but limited for apical wall evaluation due to echo signal attenuation. In contrast, ICE from the RV allows real‐time assessment of apical thickness and pericardial effusion, providing additional safety advantages. In particular, for screw‐type LP, the depth of myocardial penetration is fixed. Therefore, assessing myocardial thickness using ICE can be useful. However, assessment using ICE should be regarded as an adjunctive tool and does not, by itself, guarantee procedural safety in all cases. Moreover, insertion of the ICE catheter into the RV itself carries a potential risk of myocardial injury; therefore, careful catheter manipulation is required. Its use is best reserved for selected situations, such as when contrast agents cannot be administered or when anatomical constraints prevent catheter orientation toward the interventricular septum.

## Conflicts of Interest

The authors declare no conflicts of interest.

## Supporting information


**Video S1:** Intracardiac echocardiography movie from the RV.

## Data Availability

The authors have nothing to report.
