# Supplementary material for: Intracardiac Echocardiography–Guided Implantation of Aveir VR Leadless Pacemaker
Source: J Arrhythm. 2026 Feb 16;42(1):e70303. doi: 10.1002/joa3.70303 (PMC12910166; doi:10.1002/joa3.70303)
Supplement: Supplementary file 1 — Video S1: Intracardiac echocardiography movie from the RV. [file JOA3-42-e70303-s001.zip › joa370303-sup-0001-Supinfo1@supplemental material legend (video) 20260105.docx]

Supplemental Video 1

Intracardiac echocardiography movie from the RV.
